# Devitalisation of human cartilage by high hydrostatic pressure treatment: Subsequent cultivation of chondrocytes and mesenchymal stem cells on the devitalised tissue

**DOI:** 10.1038/srep33747

**Published:** 2016-09-27

**Authors:** B. Hiemer, B. Genz, A. Jonitz-Heincke, J. Pasold, A. Wree, S. Dommerich, R. Bader

**Affiliations:** 1 Rostock University Medical Center, Department of Orthopaedics, Biomechanics and Implant Technology Research Laboratory, Doberaner Strasse 142, 18057 Rostock, Germany; 2 Rostock University Medical Center, Department of Otorhinolaryngology, Doberaner Strasse 137–139, 18057 Rostock, Germany; 3 Rostock University Medical Center, Department of Anatomy, Gertrudenstraße 9, 18057 Rostock, Germany; 4 Charité Berlin University Medical Center, Department of Otorhinolaryngology, Chariteplatz 1, 10117 Berlin, Germany

## Abstract

The regeneration of cartilage lesions still represents a major challenge. Cartilage has a tissue-specific architecture, complicating recreation by synthetic biomaterials. A novel approach for reconstruction is the use of devitalised cartilage. Treatment with high hydrostatic pressure (HHP) achieves devitalisation while biomechanical properties are remained. Therefore, in the present study, cartilage was devitalised using HHP treatment and the potential for revitalisation with chondrocytes and mesenchymal stem cells (MSCs) was investigated. The devitalisation of cartilage was performed by application of 480 MPa over 10 minutes. Effective cellular inactivation was demonstrated by the trypan blue exclusion test and DNA quantification. Histology and electron microscopy examinations showed undamaged cartilage structure after HHP treatment. For revitalisation chondrocytes and MSCs were cultured on devitalised cartilage without supplementation of chondrogenic growth factors. Both chondrocytes and MSCs significantly increased expression of cartilage-specific genes. ECM stainings showed neocartilage-like structure with positive AZAN staining as well as collagen type II and aggrecan deposition after three weeks of cultivation. Our results showed that HHP treatment caused devitalisation of cartilage tissue. ECM proteins were not influenced, thus, providing a scaffold for chondrogenic differentiation of MSCs and chondrocytes. Therefore, using HHP-treated tissue might be a promising approach for cartilage repair.

Large populations of patients suffer from degenerative disorders of the musculoskeletal system, thus increasing the demand for novel therapeutic approaches. Especially the regeneration of cartilage lesions represents an important challenge for orthopaedic surgery. Articular cartilage has a limited innate ability for healing and self-regeneration due to the lack of vascularisation and innervation^1^. Joint injuries with cartilage defects predispose to tissue degeneration with subsequent development of osteoarthritis^2^. Numerous strategies have been investigated to repair cartilage lesions of different size, location and depth to regenerate them with biochemical and biomechanical equivalent tissue^3^. Clinical approaches include cell-based therapies aimed at the reconstruction and regeneration of cartilage tissue by transplantation of suspended chondrocytes (autologous chondrocyte implantation - ACI) or a cell/scaffold complex (matrix-associated autologous chondrocyte transplantation - MACT). Another surgical option is the use of osteochondral autografts, which means the implantation of cylindrical osteochondral grafts taken from non-load bearing regions of the articular cartilage^4^,^5^,^6^. All mentioned treatment techniques use mostly autologous tissue. Although these methods afford advantages such as the lack of tissue rejection, some serious drawbacks are apparent that limit clinical utility. Harvesting autologous tissue could result in unwanted donor side morbidity and additional long-term complications. Moreover, the availability of healthy tissue is restricted and, in the case of ACI, a second operative procedure is required^4^,^7^.

The use of allogenic cartilage could provide an alternative treatment option^8^. This is a restorative procedure in which either chondrocytes or articular cartilage and its underlying subchondral bone obtained from a donor of the same species are transplanted into the defect. Especially, osteochondral allograft transplantation has revealed encouraging results and a high success rate in clinical and experimental studies^3^. The use of allogenic material from healthy donors includes the advantage that a large amount of unimpaired tissue is available which is able to form high-quality cartilage^9^. Obviously, possible immunological tissue rejection has to be considered; however, the transplantation of decellularised cartilage matrix massively reduces the extent of immunological reaction because of the absence of cellular components^3^. Previously used decellularisation and sterilisation procedures include chemical detergents, autoclaving and irradiation. However, these techniques influence the hydration status and three-dimensional orientation of proteins, resulting in altered biomechanical properties of the cartilage tissue^10^. Therefore, to overcome these limitations, a novel approach has been developed using high hydrostatic pressure (HHP) to achieve the devitalisation of cartilage tissue while maintaining the biomechanical characteristics^11^,^12^.

HHP treatment is widely used in the food industry to inactivate microorganisms without affecting flavour, vitamin content and aroma^13^. It is considered that the effect of HHP is mediated by the entry of water molecules into proteins and subsequent destruction of the tertiary and quaternary structures. Nevertheless, covalent molecular bonds remain intact^13^. In recent years, the potential of HHP for medical applications was investigated aiming at the inactivation of pathogens to minimise the risk of infection during the transplantation of autologous and allogeneic tissue^14^. Furthermore, the HHP treatment of restricted tumour-infiltrated tissue is thought to destroy malignant cells in order to enable the reimplantation of the treated tissue as an autologous graft^14^,^15^. Different studies have already shown that HHP is able to damage both normal eukaryotic cells and malignant osteosarcoma as well as fibrosarcoma cells^11^,^15^,^16^.

HHP offers a promising approach for tissue engineering to provide devitalised cartilage allografts for reconstruction after lesion. The effects of HHP treatment on the biomechanics of different tissues and its inactivating effect on eukaryotic and prokaryotic cells has already been studied^15^,^17^,^18^. However, the devitalising impact of HHP on chondrocytes within the impervious cartilage matrix and subsequent revitalisation of devitalised cartilage has not been examined. In the present study, the capability of HHP treatment to generate allografts through the devitalisation of cartilage tissue was investigated. Therefore, the effect of HHP on the viability of chondrocytes within a cartilage matrix was tested and the potential of devitalised tissue for revitalisation using chondrocytes and mesenchymal stem cells was analysed.

## Results

### Effect of HHP treatment on chondrocyte viability

**Figure 1:**
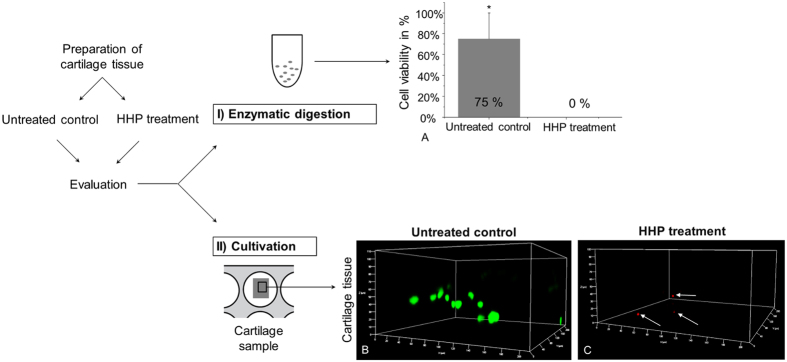
Investigation of cell viability after HHP treatment. (**A**) After tissue preparation, HHP treatment and subsequent digestion, trypan blue exclusion test indicated no viable chondrocytes in HHP-treated cartilage in contrast to untreated controls (n = 5, mean ± SD, *p < 0.05). (**B**,**C**) Live/dead staining of the cartilage matrix was analysed by confocal laser scanning microscopy and showed green fluorescent, viable cells in untreated controls compared to HHP-treated samples which displayed only red fluorescent, dead cells (white arrow).

After enzymatic digestion of HHP-treated and untreated cartilage tissue, the trypan blue exclusion test indicated cell viability of 75% ± 25% for untreated controls (Fig. 1A). However, pressure-loaded samples contained no viable cells (cell viability: 0%). Afterwards, digested cartilage samples were cultured for seven days in complete cell culture medium. Untreated controls contained adherent and viable cells which were determined by live/dead staining. Whereas untreated controls showed a high number of viable (green fluorescent) cells after cultivation, for HHP-treated cartilage no living cells were detected (data not shown).

Additionally, HHP-treated and untreated cartilage segments remain undigested and were cultured in complete cell culture medium. Using laser scanning microscopy, live/dead staining indicated viable cells within the cartilage tissue of control samples after 28 days (Fig. 1B). In the HHP-treated cartilage samples, no viable chondrocytes could be detected (Fig. 1C). During cultivation, the human chondrocytes grew out of the untreated cartilage samples and proliferated on the tissue culture polystyrene (TCP), while no outgrowth of cells from devitalised samples could be observed after HHP-treatment (data not shown).

### Cellular alterations induced by HHP treatment

**Figure 2:**
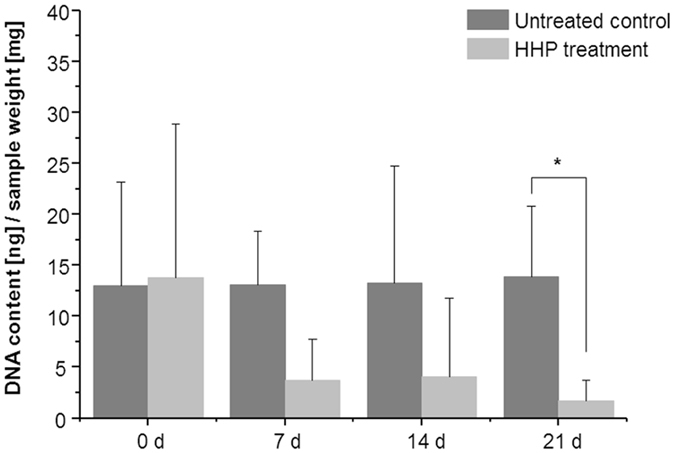
Ratio of DNA content related to sample weight of HHP-treated cartilage and untreated controls. After 21 days of cultivation, the ratio was significantly decreased for HHP-treated cartilage compared to untreated controls. After HHP treatment, no sign of proliferation and viable cells could be detected within cartilage matrix (n = 5, mean ± SD, *p < 0.05).

HHP-induced cell inactivation is accompanied by the degradation of DNA. The total DNA amount was quantified and normalised to the respective sample weight (Fig. 2). Immediately after HHP treatment, control and treated samples presented similar ratios of DNA/sample weight. Vital cartilage (untreated controls) showed a high DNA/sample weight ratio, which was constant up to 21 days of cultivation, whereas the ratio of HHP-treated cartilage was significantly decreased after 21 days (p = 0.029) compared to the control.

The quality of the sample RNA provides information regarding molecular alterations induced by HHP. Spectrophotometric analysis of RNA samples showed a significantly reduced A260/A280 ratio after HHP treatment compared to untreated cartilage (p = 0.046), indicating degraded RNA with poor quality. The isolated RNA of untreated controls provided an A260/A280 ratio of 1.898 ± 0.164, indicating pure and intact RNA (Table 1).

### Effect of HHP on cartilage ECM

**Figure 3:**
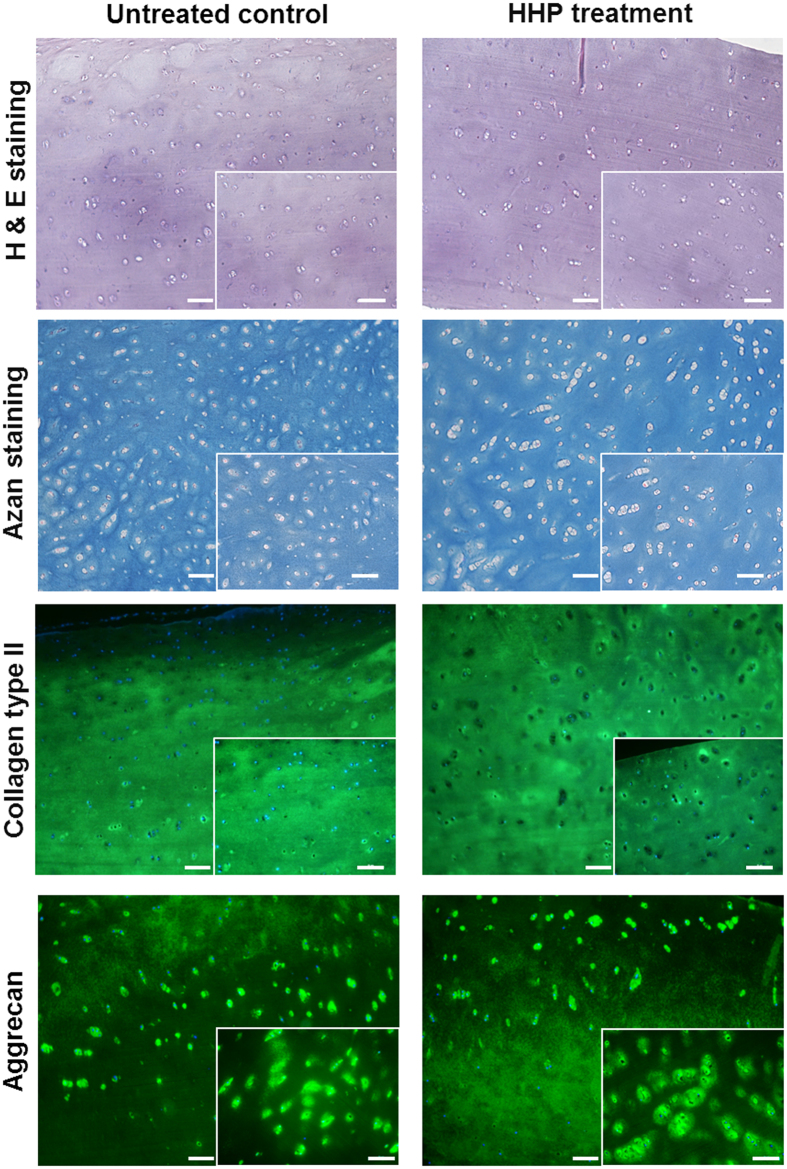
Histological and immunohistochemical staining of HHP-treated cartilage and untreated controls. Stainings were performed 14 days after HHP treatment. The first panel shows staining with haematoxylin and eosin (H&E). Using H&E cell nuclei were stained blue and cell cytoplasm as well as extracellular proteins were stained bright red. Second panel displays the cartilage-specific Heidenhain’s AZAN trichrome staining whereby collagen is stained blue and cell nuclei red. The panels below demonstrated the immunohistological stainings with collagen type II and aggrecan antibodies fluorescing in green. Counterstaining was performed with Hoechst 33342 visualising cell nuclei in blue. Alterations in ECM staining between HHP-treated and untreated cartilage could not be detected (low magnification: bar = 100 μm; high magnification: 50 μm).

Alterations in the structure of cartilage extracellular matrix (ECM) were investigated using histological and immunohistochemical staining as well as FESEM analyses. For an overview of cell morphology and ECM haematoxylin and eosin (H&E) as well as Heidenhain’s AZAN trichrome staining were performed (Fig. 3). The histological stainings exhibited intact cartilage tissue in both pressure-loaded and control samples without any lesions or destructions. The staining and dyability of extracellular proteins remained unchanged. Immunohistochemical staining of the prominent ECM components were conducted to observe shifts in protein conformation. Figure 3 shows that collagen type II and aggrecan could be detected even after HHP treatment. However, the cell nuclei stained using Hoechst 33342 shined brighter in untreated controls compared to cell nuclei of HHP-treated tissue indicative for a successful devitalisation.

**Figure 4:**
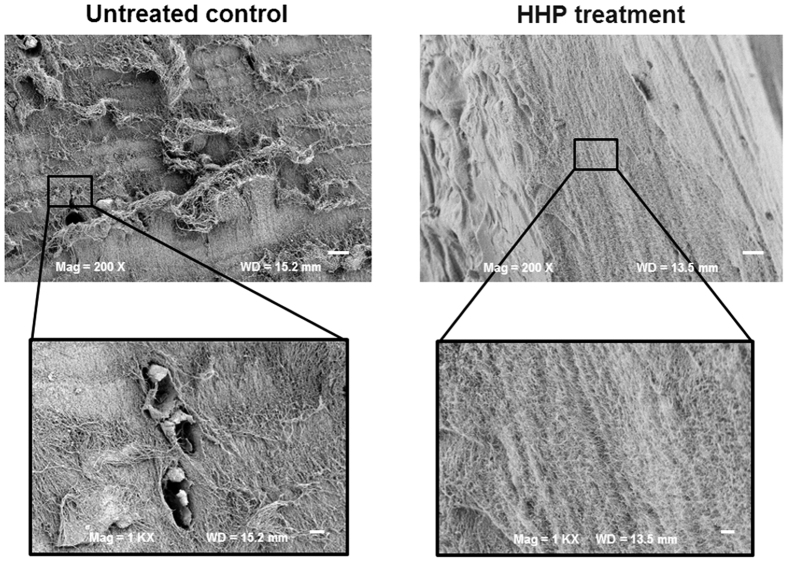
FESEM images of untreated and HHP-treated cartilage. High pressure treatment did not affect the ECM structure of hyaline cartilage (upper panel: bar = 20 μm; lower panel: bar = 5 μm).

Moreover, FESEM analyses demonstrated no damaged areas in HHP-treated tissue, indicating the preserved integrity of the tissue (Fig. 4). The collagen fibres of pressure-loaded cartilage were in the native conformation and showed no signs of disruption or damage.

### Using devitalised cartilage tissue as scaffold for the cultivation of chondrocytes and MSCs

In order to verify the possible application of HHP in the field of regenerative medicine, devitalised cartilage tissue was used as a scaffold for the cultivation of human chondrocytes and mesenchymal stem cells derived from adipose tissue (AD-MSCs) as well as bone marrow (BM-MSCs). The tissue biocompatibility was examined using chondrocytes. Live/dead staining indicated various viable (green fluorescent) cells on the tissue surface (data not shown).

**Figure 5:**
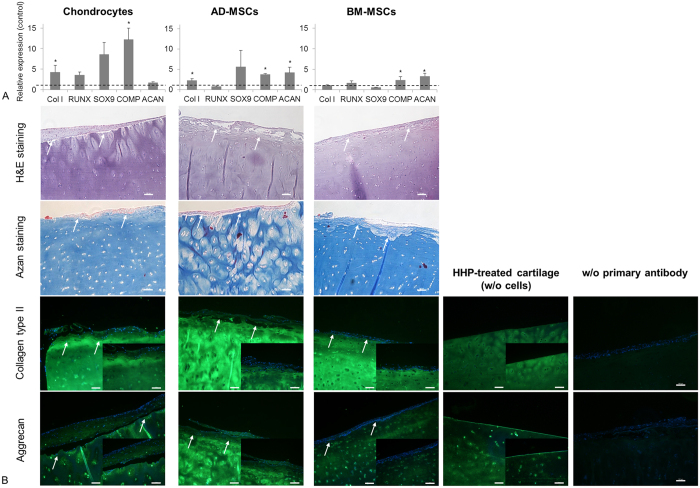
Cultivation of chondrocytes and MSCs onto devitalised cartilage. Cells were cultured for 21 days onto devitalised cartilage in cell culture medium supplemented with ascorbic acid. (**A**) Chondrogenic differentiation was examined using gene expression analyses of *Col I* (collagen type I), *RUNX* (Runt-related transcription factor 2), *SOX9* (Sry-related HMG box 9), *COMP* (cartilage oligomeric matrix protein) and *ACAN* (aggrecan) after 21 days of cultivation. Data are normalised to the respective controls (AD-MSCs, BM-MSCs and chondrocytes, respectively) cultured in fibrin glue on TCP. All three cell types show significantly increased expression rates of cartilage-specific genes compared to control (mean ± SD, n = 3, *p < 0.05). (**B**) Histological and immunohistological staining of cells (chondrocytes and MSCs) cultured onto devitalised cartilage to investigated chondrogenic differentiation. H&E staining were performed to get an overview of cultured cells. Heidenhain’s AZAN trichrome staining of cell/devitalised cartilage constructs indicated neocartilage-like ECM synthesised by AD-MSCs, BM-MSCs and chondrocytes. Collagen is stained blue and cell nuclei red. Immunohistochemical staining of collagen type II and aggrecan indicated a differentiation into chondrogenic lineage. Arrows display the crossover from cartilage tissue to cell layer. HHP-treated cartilage tissue where no cells were seeded on shows no sign of viable cells. This indicates that no vital cells could grow out of the cartilage. At the right site controls of antibody staining are provided (low magnification: bar = 100 μm; high magnification: 50 μm).

For application as a biomaterial, the influence of devitalised cartilage on the differentiation of MSCs has to be considered as well as the potential to redifferentiate chondrocytes which dedifferentiated while expansion in monolayer (Fig. 5A). Gene expression analyses demonstrated that *SOX9*, an essential transcription factor of chondrogenic differentiation, is upregulated in chondrocytes and AD-MSCs but not for BM-MSCs. However, both AD-MSCs and BM-MSCs cultured on devitalised cartilage showed significantly increased expression of the chondrogenic markers *COMP* (p = 0.037 (AD-MSC); p = 0.037 (BM-MSCs)) and *aggrecan* (p = 0.037 (AD-MSC); p = 0.046 (BM-MSCs)) compared to respective controls cultured in fibrin glue on TCP. Using BM-MSCs the *collagen type I* expression as an indicator of unwanted fibrocartilage formation was not increased. In chondrocytes, *COMP* expression was significantly increased (p = 0.037), pointing to initial redifferentiation of the cells. *Collagen type I* expression was also significantly increased in chondrocytes (p = 0.046) and even in AD-MSCs (p = 0.037). The expression of transcription factor *RUNX-2* as a hypertrophy marker was not increased. Additionally, the devitalised cartilage without seeded cells were analysed as control. Thereby, Ct-values were similar to the negative control (DEPC-water) indicating an absence of gene expression.

Gene expression analyses were confirmed by histological and immunohistochemical stainings (Fig. 5B). Stainings (H&E and AZAN) showed that after 21 days of cultivation cells seeded on top of the devitalised cartilage covered the tissue surface densely. AZAN staining detected neocartilage-like matrix indicated by a blue staining of collagen. Using chondrocytes for revitalisation, an enhanced amount of newly synthesized collagen type II and aggrecan was revealed. Staining with aggrecan antibody and Hoechst 33342 did detect chondrocytes very close to the devitalised cartilage matrix and might infiltrate the upper layer of chondrons. Using AD-MSCs and chondrocytes the immunohistochemical staining of collagen type II and aggrecan was more intense compared to BM-MSCs. However, results indicated that both chondrocytes and MSCs synthesised more aggrecan than collagen type II. After HHP treatment the devitalized cartilage without any seeded cells showed no sign of viable cells in both the inside and outside of the tissue. Analysing the staining without primary antibody, it is remarkable that the seeded cells on the cartilage surface shined brightly, however, within the devitalised cartilage signals could be detected barely, confirming the successful devitalisation.

## Discussion

Once the development of hyaline cartilage is completed, chondrocytes show no or limited potential for self-regeneration after surface damage^19^. Therefore, the healing process represents a major challenge in orthopaedic surgery. There are numerous treatment strategies for cartilage tissue injuries, including cell- and scaffold-based methods. Nevertheless, long-term studies point to major concerns about the production of inferior fibrocartilage, which is unable to withstand the same biomechanical loading as the surrounding healthy hyaline cartilage^3^,^20^,^21^.

For cartilage regeneration, synthetic scaffolds are used to provide a three-dimensional environment supporting initial cell attachment, proliferation and differentiation. However, an adequate mimic of the physiological structure has not been achieved yet. Therefore, a novel concept in tissue engineering is the use of cartilage ECM, because of its natural regulatory molecules and mechanical stability, which could be capable of enhancing regeneration processes. This cartilage matrix can be created by different devitalising processes, which include the inactivation of viable cells or decellularising procedures which involve the cell inactivation and the additional removal of nearly all cells and residual cellular components^22^.

In the present study, high hydrostatic pressure (HHP) was used for the devitalisation of cartilage tissue. Live/dead staining of pressure-loaded tissue could not detect any living cells within the cartilage matrix and no outgrowth of cells occurred. The results were confirmed by the trypan blue exclusion test. HHP-treated samples contained no viable cells, in contrast to controls representing a high number of living cells. These results are in agreement with the study by Naal *et al*. outlining the destructive impact of HHP^15^; pressure in the range of 350 MPa causes the inactivation of all human chondrocytes and chondrosarcoma cells (SW1353) in adherent as well as suspended status. HHP-induced cell death has also been investigated in the human osteosarcoma cell line Saos-2 and the fibrosarcoma cell line HT-1080^23^.

During cell death, DNA and other cellular organelles are eliminated. Our investigations show a significant degradation of chondrocyte DNA after HHP treatment. However, different studies have revealed that HHP does not influence intermolecular hydrogen bonding indicating HHP-resistant properties of DNA^13^,^18^. Therefore, we conclude that the DNA degradation is not a primary result of HHP treatment but is rather related to succeeding processes during cell death induced by HHP. DNA elimination seems to be delayed, which could be a result of the dense cartilage ECM and impaired transport of cellular compounds^10^. The DNA content is a criterion for assessing the efficiency of cell inactivation concerning DNA levels below 50 ng/mg tissue weight^24^. This guideline was satisfied by devitalised cartilage 21 days after HHP treatment. After pressure loading, no sign of proliferation in terms of increased DNA content was detectable, indicating no living cells within the tissue. The detection of cell nuclei within devitalized tissue using Hoechst binding to DNA is clearly reduced. Assessments of RNA integrity showed that HHP caused damage to fundamental elements of cell metabolism and protein synthesis, resulting in cell death.

In contrast to devitalisation using HHP, the common decellularisation of cartilage involves extensive chemical, physical and enzymatic processes^22^. Because of the dense structure of cartilage, the exposure time to chemical agents has to be increased, leading to a higher degree of ECM destruction^25^,^26^ and the loss of some of its biomechanical stability^26^,^27^,^28^. Different studies have determined the importance of preserving the ECM structure to induce the preferred regenerative effect^26^,^29^. This indicates the relevance of cell inactivation techniques which cause reduced alterations in the architecture and stability of the treated tissue. The benefit of using HHP treatment is that compounds of cartilage ECM appears to be unaffected. Histological staining could not identify any damage or destruction of the cartilage matrix. The staining and dyability of the tissue remained unchanged indicative for maintaining physical properties of HHP-treated ECM proteins like macromolecule charge. Antibodies against collagen type II and aggrecan were used to visualise major components of cartilage ECM to detect possible matrix alterations. Immunohistochemical staining is specific, and can reveal minimal changes within protein molecules. The comparison between HHP-treated cartilage and untreated controls demonstrated no significant differences. Additional FESEM analyses showed no sign of alterations in the alignment and structure of collagen fibres. Investigations using cartilage and bone have demonstrated that tissue integrity was maintained after HHP treatment^30^,^31^. Moreover, some studies have revealed that HHP does not change the biomechanical properties of articular cartilage^16^,^32^, which may have a positive influence on the development of superior neotissue and enables the ECM to respond on joint loading and motion^10^,^29^.

HHP treatment results in the inactivation of living cells, while retaining the cartilage microstructure and, therefore, represents a promising devitalisation approach for tissue engineering. Devitalised cartilaginous matrix may better integrate into the surrounding tissue because of its similar structure^22^. Tissue obtained from human cadavers or a xenogeneic source would be desirable due to the need for only a single surgical procedure for tissue transplantation and the absence of donor site morbidity related to autologous graft harvesting^8^. Considering above mentioned benefits, HHP-treated cartilage tissue is assumed to be an appropriate biomaterial for allogenic restoration of osteochondral defects.

However, using HHP-treated tissue as scaffold for allogenic transplantation, its immunogenicity has to be considered. The treatment of cartilage using HHP causes a devitalisation only. This means that DNA and other cellular residuals remain within the cartilage tissue. Cell surface markers, ECM epitopes and residual DNA could arise an immune response from host to graft tissue^22^. Although chondrocytes and their synthesised ECM are able to activate the immune system^3^,^10^, the avascularity of cartilaginous tissue could impart a certain degree of immunoprivileged properties of that tissue^10^. Additionally, the impervious network of ECM proteins prevents contact between graft antigens and host immune response^3^. Corresponding to that, it has been figured out that transplantation of suspended chondrocytes evoke an immune response, whereas, osteochondral allografts provoke an acceptable immunological response^3^. Moreover, in contrast to decellularisation processes and conservative devitalisation techniques like cryopreservation and freeze-drying, HHP treatment performed a devitalisation which is gently for the tissue. The dense ECM network remains intact and, therefore, immune system relevant cells of the host are separated from allogenic chondrocytes of deeper ECM zones. Fresh osteochondral allografts which means allografts without any removal of cells and cellular residuals have already demonstrated use in a wide spectrum of knee joint pathology^33^,^34^. Investigations regarding immunologic response show a postallograft antibody formation related to graft size. However, differences in the graft survival rates between antibody-positive and antibody-negative groups did not reach level of significance^35^. In addition, cartilaginous allografts have revealed clinical success in the treatment of focal femoral condyle lesions in more than 75% of the cases^36^. However, HHP treatment maintained cartilage tissue structure while inactivate cells, resulting in a reduction of immunological risks.

Another important point for the use of HHP-treated cartilage in the field of regenerative medicine is the potential for revitalisation. Since ECM characteristics are retained, devitalised cartilage constructs reflect the biological complexity and mimic both the physiological microenvironment and the three-dimensional structure of the cartilage matrix, which could induce chondrogenic differentiation^37^,^38^. Gene expression analyses and stainings demonstrated the potential of HHP-treated cartilage to direct MSCs into the chondrogenic lineage. SOX9 is identified as a key transcription factor for chondrogenic differentiation and essential for collagen type II and aggrecan synthesis^39^. Gene expression analyses demonstrated an upregulation of *SOX9* in chondrocytes cultured on devitalized cartilage. Additionally, mRNA of *COMP* and *aggrecan* were significantly increased. These findings are in accordance with positive immunhistochemical staining of collagen type II and aggrecan as well as AZAN staining indicating neocartilage-like structures synthesised by MSCs and chondrocytes. For chondrogenic differentiation, growth factors like TGF-β3, FGF and IGF-1 are essential^40^. In the present study, MSCs were cultured on the devitalised ECM using cell culture medium supplemented with ascorbic acid, but without the addition of growth factors. Nevertheless, MSCs showed significantly increased chondrogenic differentiation compared to MSCs cultured in fibrin glue on TCP. Therefore, additional factors have to be considered which are present within the devitalised cartilage matrix^10^,^41^. Retention of bioactive and regulatory molecules would be beneficial for regeneration processes, since the vascular supply is limited in this tissue and, therefore, growth factor availability is restricted^10^,^38^.

Results indicated that MSCs and chondrocytes seeded on the HHP-treated cartilage differentiate into chondrogenic lineage. The proliferation on the surface was detected demonstrating that HHP-treated tissue is biocompatible. However, the migration into deeper zones of cartilage could be impaired. Histological staining and FESEM analyses revealed the dense ECM structure of cartilage tissue complicating a cell migration through the ECM. Matrix metalloproteinases are enzyme which degrades triple-helical collagen structure and realizes turnover and remodelling of cartilage ECM^42^. An enhanced expression of MMPs may result in break up of dense ECM structure and enables a migration of cells in deeper zones of cartilage. However, an upregulated expression of MMP 1 mRNA in MSCs and chondrocytes seeded on devitalised cartilage could not be detected (data not shown). However, the *in vitro* cultivation could not mimic the physiological conditions entirely. Therefore, the revitalisation of HHP-treated tissue will be investigated in further *in vivo* animal studies. In case of using HHP-treated tissue as scaffold for cells in terms of cartilage regeneration, the residual dead cells have to be considered. The effect of that inactivated cells on proliferation, apoptosis and migration of seeded cells could not be investigated *in vitro*. Further *in vivo* studies will explain whether a migration of cells through the HHP-treated tissue will occur and how cells interact with the devitalised ECM. Successful *in vivo* revitalisation of decellularised tissue has been performed by Benders *et al*.^10^ who investigated osteochondral defects in the knee joints of horses. The treatment was based on the use of decellularised cartilage and presented clear regeneration in both cartilage and bone segments after 8 weeks. Another study show that acellular cartilage matrix led to improved repair of knee defects compared to the control group after six and 12 weeks in a rabbit model^27^. For *in vivo* investigations of the revitalisation and integration of HHP-treated tissue (osteochondral cylinders) animal studies will be performed on the knee of New Zealand White rabbits. Additionally, biomechanical properties of HHP-treated osteochondral cylinders implanted *in vivo* have to be tested to verify tissue stability of repair tissue.

## Conclusion

Using devitalised ECM, major problems associated with the regeneration of cartilage injuries could be solved. Cartilage represents a suitable tissue for devitalisation because this tissue contains only one cell type and no vascular, lymphatic or nerve structures^2^. Our results suggest that HHP treatment could be used for the devitalisation of human cartilage tissue while maintaining ECM structure. Moreover, even after HHP treatment, cartilage presents bioactive cues to promote chondrogenic differentiation of MSCs and dedifferentiated chondrocytes. In further *in vivo* studies the possible clinical application has to be proven. The integration of HHP-treated tissue to the original, surrounding cartilage will be investigated and also the related regenerative processes. HHP-induced devitalised cartilage may provide a promising scaffold material for cartilage regeneration.

## Materials and Methods

### Sample preparation

Human articular knee cartilage was obtained from patients undergoing primary knee replacement (n = 7, male: 64 ± 12 years; female: 62 ± 14 years). The samples were only collected with patient informed consent and according to procedures approved by the Local Ethical Committee of the University of Rostock (registration number: A2009 17). Subsequent treatments were also carried out in accordance with these approved guidelines. The hyaline cartilage was cut into equal samples with a size of 0.5 cm × 1 cm under sterile conditions. The specimens were cultured in cell culture medium consisting of Dulbecco’s Modified Eagle Medium (DMEM, Invitrogen, Darmstadt, Germany) containing 10% foetal calf serum (FCS), 1% amphotericin B and 1% penicillin-streptomycin under standard cell culture conditions (5% CO_2_ and 37 °C). For the induction of chondrogenic differentiation, the medium was supplemented with ascorbic acid (50 μg/ml, Sigma, Seelze, Germany) and will be further referred to as complete cell culture medium.

### High hydrostatic pressure treatment

**Figure 6:**
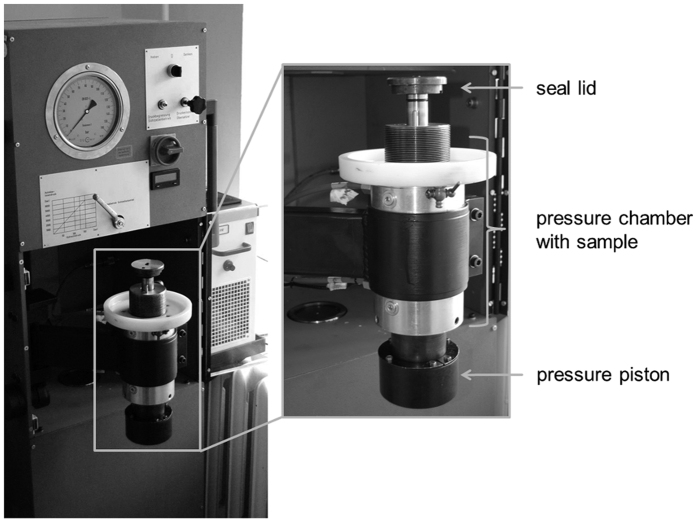
High hydrostatic pressure device HDR-100 for devitalisation of cartilage tissue. Samples were placed in the hermetically sealed pressure chamber and treated for 10 min at 480 MPa.

The parameters chosen for HHP treatment based on previous studies involving investigations using articular and meniscal cartilage and ossicles^11^,^12^,^18^,^30^. The devitalisation of cartilage segments was performed 24 to 48 h after sample preparation using HHP. Samples were placed in 2 ml- cryo-tubes completely filled with cell culture medium to avoid air bubbles. Additionally, the vials were sealed with parafilm. The tubes were placed into the glycol-filled pressure chamber of the HHP device (HDR-100, RECORD GmbH, Koenigsee, Germany) and treated for 10 min at approximately 480 MPa and 20 °C (Fig. 6). Untreated cartilage samples were equally handled and served as untreated control.

### Analysis of the devitalising effect of HHP treatment

#### Cell viability - trypan blue exclusion test

Immediately after decompression, human HHP-treated cartilage samples and corresponding controls were cut into small pieces and washed with phosphate buffered saline (PBS; PAA, Coelbe, Germany). Subsequently, the samples were digested with 1% trypsin/EDTA (Gibco Invitrogen, Darmstadt, Germany) for 20 min at 37 °C followed by treatment with 0.2% collagenase A (Roche, Mannheim, Germany) mixed with cell culture medium overnight at 37 °C. Afterwards, each digested cartilage sample was checked for cell viability by the trypan blue exclusion test. The cell suspension was mixed with trypan blue dye (Serva, Heidelberg, Germany) which enters cells with damaged membranes to highlight dead cells. Viable cells remain unstained. Cells were examined using a hemocytometer and inverted microscope (Televal 31, Carl Zeiss, Jena, Germany). The number of blue stained (dead) cells and the total number of cells were counted. Cell viability was calculated using the following formula:

% cell viability = (number of viable cells/number of total cells) × 100.

#### Cell viability - live/dead staining

Cartilage segments (pressure-loaded and untreated samples) were cultured in complete cell culture medium for up to 28 days. Subsequently, live/dead staining was performed using a LIVE/DEAD assay kit (Life Technologies, Carlsbad, USA). The two-colour assay discerns living from dead cells by simultaneously staining with green fluorescent (494–517 nm) calcein-acetoxymethyl, indicating intracellular esterase activity, and red fluorescent (528–617 nm) ethidium homodimer-1, showing the loss of plasma membrane integrity. The assay was performed according to the manufacturer’s instructions. Images were taken with confocal laser scanning microscopy (LSM 780, Carl Zeiss, Jena, Germany).

#### Purification of total DNA

The DNA content of cartilage samples was quantified using a DNeasy Blood and Tissue Kit (QIAGEN, Hilden, Germany) according to the manufacturer’s specifications and DNA concentration was measured with a spectrophotometer (Infinite 200 PRO, TECAN, Maennedorf, Switzerland). Samples were weighed prior to lysis and the DNA content was related to the respective sample weight. DNA was purified directly after HHP treatment (day 0). Additionally, DNA content of HHP-treated samples and untreated controls was quantified after cultivation for 7, 14 and 21 days.

#### Integrity of sample RNA

RNA integrity was investigated as indicator of cell damage after HHP treatment. Samples were digested using proteinase K (0.5 mg/ml in Tris-HCl; Peqlab, Erlangen, Germany). Afterwards, the isolation of RNA was performed using the Direct-zol RNA MiniPrep Kit (Zymo Research, Freiburg, Germany) according to the manufacturer’s protocol. The RNA concentration and purity were determined by measuring the absorbance (A) at 260 nm and 280 nm with a spectrophotometer (Infinite 200 PRO, TECAN, Maennedorf, Switzerland). The A260/A280 ratio was used to evaluate RNA quality. Values between 1.8 and 2.0 indicated high purity and low protein content.

### Effect of HHP treatment on structural integrity

#### Histological and immunohistochemical staining

Histological staining was performed to examine the alterations to the extracellular matrix (ECM). Pressure-loaded human cartilage and untreated samples were fixed in 4% buffered formalin for 24 h, embedded in paraffin and subsequently cut into 5 μm thick sections. These sections were deparaffinised and stained with either hematoxylin (H) and eosin (E) or Heidenhain’s AZAN trichrome^43^,^44^. Pictures were taken with a light microscope (Nikon Type 120, Nikon Instruments, Tokyo, Japan).

For immunostaining, slices were pretreated for 30 min at 37 °C with pronase (1 mg/ml in PBS, Roche Applied Sciences, Mannheim, Germany) as well as hyaluronidase (10 mg/ml in PBS + 0.01% bovine serum albumin (BSA), Sigma, Seelze, Germany) for collagen type II antibody or with chondroitinase ABC (5 U/ml in PBS + 0.01% BSA, Sigma, Seelze, Germany) and Endo-β-galactosidase (5 U/ml in PBS + 0.1% BSA, Sigma, Seelze, Germany) for aggrecan antibody. Afterwards, the cartilage sections were incubated with the primary antibodies anti-chicken collagen type II (1 μg/ml, Chemicon Int., Atlanta, USA) or anti-human aggrecan (2.5 μg/ml, R&D Systems, Minneapolis, USA) overnight at 4 °C. The secondary antibody goat-anti-mouse-Alexa Fluor 488 (0.01 mg/ml, Molecular Probes, Darmstadt, Germany) was incubated for 1 h at room temperature. Non-specific binding of the secondary antibodies was assessed by omitting the primary antibody. Furthermore, all slides were counterstained with Hoechst 33342 (5 μg/ml, Enzo Life Sciences, Loerrach, Germany). Pictures were taken with a fluorescence microscope (Nikon Type 120, Nikon Instruments, Tokyo, Japan).

#### Electron microscopy

Field emission scanning electron microscopy (FESEM) analyses were used to evaluate structural modifications to the HHP-treated cartilage tissue and corresponding controls. Hence, cartilage segments were cut across for cross-sectional investigation. The samples were rinsed with PBS and fixed in 2% glutaraldehyde buffer. Afterwards, the samples were washed with PBS twice prior to dehydration through a graded series of ethanol. Critical point drying (EMITECH K850, Ashford, Great Britain) was performed and a uniform layer of gold was applied. FESEM viewing was conducted using a MERLIN VP Compact microscope (Carl Zeiss, Jena, Germany) operated at an accelerating voltage of 5 kV. ECM structure was investigated on five random low power fields per sample.

### Cultivation of cells onto HHP-treated cartilage

#### Cell cultivation onto devitalised cartilage

Revitalisation of devitalised cartilage was analysed with human adipose-derived MSCs (AD-MSCs) and bone marrow-derived MSCs (BM-MSCs), acquired from ATCC LGC Standards GmbH (Wesel, Germany). Cells were expanded up to passage five in the recommended MesenPRO RS medium (Life Technologies, Carlsbad, USA) before seeding onto the devitalised samples.

Additionally, human chondrocytes were used to investigate the revitalisation of the devitalised cartilage tissue. The dissected cartilage was divided into two parts. One fraction was prepared for HHP treatment as mentioned above (section: sample preparation) and the second part of the cartilage tissue was used to isolate cells according to the previously described protocol^45^. Isolated chondrocytes were expanded up to passage three and subsequently seeded onto devitalised cartilage from the same donor.

For cell application (MSCs and chondrocytes), a fibrin glue (Baxter, Berlin, Germany) was used, consisting of thrombin diluted in a ratio of 1:1000 with cell culture medium and fibrinogen solution diluted 1:4 with cell culture medium. After 45 min incubation at 37 °C, medium was added and the samples were cultured for 21 days in cell culture medium supplemented with ascorbic acid. MSCs and chondrocytes also cultured in fibrin glue on TCP served as control.

#### Gene expression analysis

The chondrogenic differentiation of the three different cell types was estimated by gene expression analyses. RNA isolation was performed as mentioned above. Single-stranded cDNA was synthesised from total RNA using the High Capacity cDNA Reverse Transcription Kit following the manufacturer’s instructions (Applied Biosystems, Forster City, USA). The synthesised cDNA served as a template for quantitative real-time PCR using the innuMIX qPCR MasterMix SyGreen (Analytik Jena AG, Jena, Germany) and specific forward and reverse primers shown in Table 2. The cycling conditions for amplification were 95 °C for 2 min, 40 cycles of 95 °C for 5 sec and 65 °C for 25 sec. The expression of all genes was normalised to the expression of the corresponding housekeeping gene (β-actin). The relative amount of target mRNA in controls and cells cultured on devitalised cartilage was expressed as 2^−(ΔΔCt)^, where ΔΔCt = ΔCt_cartilage_ − ΔCt_control_.

### Statistical analysis

Data are represented as the mean value ± standard deviation (SD). At least three independent experiments were performed for statistical analysis. Statistical significance between groups was calculated by non-parametric Mann-Whitney U test using SPSS Statistics 2.0 (IBM, Ehningen, Germany). The level of significance was set to p < 0.05.

## Additional Information

**How to cite this article**: Hiemer, B. *et al*. Devitalisation of human cartilage by high hydrostatic pressure: Subsequent cultivation of chondrocytes and mesenchymal stem cells on the devitalised tissue. *Sci. Rep.* **6**, 33747; doi: 10.1038/srep33747 (2016).

## Figures and Tables

**Table 1:** Spectrophotometric analysis at absorbance (A) at 260 nm and 280 nm.

	RNAconcentration	Absorbance (A)		
		A 260 nm	A 280 nm	Ratio A 260/A 280
Untreated control	75.15 ± 26.29	0.091 ± 0.037	0.048 ± 0.016	1.84 ± 0.164
HHP treatment	7.59 ± 3.78	0.009 ± 0.005	0.0107 ± 0.006	0.96 ± 0.141*

The A260/A280 ratio was used to determine the RNA purity of the samples. A pure RNA sample has an A260/A280 ratio of 1.8–2.0^#^.

^#^n = 3, mean ± SD, *p < 0.05 versus untreated control.

**Table 2:** Sequences of cDNA primer used for qRT-PCR.

Gene	Direction	Primer nucleotide sequence
β-actin	Forward	5′-CTTCCTGGGCATGGAGTC-3′
	Reverse	5′-AGCACTGTGTTGGCGTACAG-3′
Aggrecan (ACAN)	Forward	5′-ACAAGGTCTCACTGCCCAAC-3′
	Reverse	5′-AATGGAACACGATGCCTTTC-3′
Cartilage oligomeric matrix protein (COMP)	Forward	5′-GTGGTGCTCAACCAGGGAAG-3′
	Reverse	5′-AAGCCCGCATAGTCGTCATC-3′
Collagen type I (*Col1A1*)	Forward	5′-ACGAAGACATCCCACCAATC-3′
	Reverse	5′-AGATCACGTCATCGCACAAC-3′
*Runt-related transcription factor 2 (RUNX-2*)	Forward	5’-CGCCTCACAAACAACCACAG-3'
	Reverse	5’-ACTGCTTGCAGCCTTAAATGAC-3'
*Sry-related HMG box 9 (SOX 9*)	Forward	5′-AGTACCCGCACCTGCACAAC-3′
	Reverse	5′-CGCTTCTCGCTCTCGTTCAG-3′
